# Determinanten der Inanspruchnahme der Krebsfrüherkennung von älteren Erwachsenen in Sachsen-Anhalt – Welchen Einfluss hat die Gesundheitskompetenz auf die Inanspruchnahme?

**DOI:** 10.1007/s00103-023-03806-0

**Published:** 2023-12-12

**Authors:** Lena Kannengießer, Ruben Ulbrich, Claudia Hasenpusch, Ilona Hrudey, Svenja Walter, Christoph Stallmann, Enno Swart, Stefanie March

**Affiliations:** 1 Institut für Sozialmedizin und Gesundheitssystemforschung, Medizinische Fakultät, Otto-von-Guericke-Universität Magdeburg (Sachsen-Anhalt), Leipziger Str. 44, 39120 Magdeburg, Deutschland; 2 Fachbereich Soziale Arbeit, Gesundheit und Medien, Hochschule Magdeburg-Stendal, Magdeburg (Sachsen-Anhalt), Deutschland

**Keywords:** Krebsvorsorge, Prävention, Ältere Erwachsene, Querschnitterhebung, Sachsen-Anhalt, Cancer screening, Prevention, Older adults, Cross-sectional study, Saxony-Anhalt

## Abstract

**Hintergrund:**

Um Gesundheit und damit gesellschaftliche Teilhabe zu erhalten, ist es für ältere Menschen bedeutsam, informierte gesundheitsrelevante Entscheidungen zu treffen, wie solche zur Inanspruchnahme einer Sekundärpräventionsleistung wie der Krebsfrüherkennungsuntersuchung (KFU). Nationale und internationale Studien zeigen, dass verschiedene Prädiktoren die KFU-Teilnahme bedingen. Ziel dieser Studie ist es, die KFU-Inanspruchnahme älterer Personen in einer strukturschwachen Region zu eruieren.

**Methoden:**

2021 wurden im Rahmen einer Querschnitterhebung in je 2 städtisch und ländlich geprägten Gemeinden Sachsen-Anhalts Personen ab 55 Jahren zu Determinanten, Gründen und Barrieren der Inanspruchnahme von Präventionsleistungen befragt (*n* = 954). Mittels binär-logistischer Regression werden Determinanten der Inanspruchnahme von KFU analysiert.

**Ergebnisse:**

Drei Viertel der Studienpopulation (76,6 %) nahmen nach eigenen Angaben mindestens einmal eine Leistung der Krebsfrüherkennung in Anspruch. Die multivariablen Analysen verdeutlichen maßgebliche Einflussfaktoren für eine Inanspruchnahme von KFU. Dazu gehören das Alter, partiell das Wissen über KFU, die KFU als Leistung eines Bonusprogramms der Krankenkasse, Erfahrungen mit Krebserkrankungen im engeren Umfeld, die Gedanken über die eigene Gesundheit sowie das sichere Gefühl, welches eine Teilnahme verleiht. Deskriptiv ist die ärztliche Empfehlung der stärkste Beweggrund für eine Teilnahme.

**Schlussfolgerung:**

Die Analysen zeigen, dass die KFU im Allgemeinen von älteren Erwachsenen in Sachsen-Anhalt gut angenommen werden, die Teilnahme an diesen aber nicht mit der Gesundheitskompetenz zusammenhängt. Im Sinne des Nationalen Krebsplans sollten ältere Personen grundsätzlich bei einer informierten Entscheidung unterstützt werden, bspw. durch zielgruppengerechte ärztliche Aufklärung.

## Abstract (en)

**Background:**

In order to preserve health and thus social participation, it is important for older people to make health-related decisions, such as those regarding the use of a secondary prevention service like cancer screening. National and international studies show that various predictors determine cancer screening participation. The aim of this study is to determine the cancer screening utilization of older people in a structurally weak region.

**Methods:**

In 2021, a cross-sectional study in each of two urban and rural communities in Saxony-Anhalt surveyed individuals aged 55 years and older about determinants, reasons, and barriers to preventive service use (*n* = 954). Binary logistic regression analysis is used to analyze determinants of cancer screening use.

**Results:**

Three quarters of the study population (76.6%) self-reported participating in a cancer screening service at least once. The multivariable analyses demonstrate factors that influence the utilization of cancer screening. Age, partial knowledge on cancer screening, cancer screening as a benefit offered by a statutory health insurances’ bonus program, experience with cancer in the immediate environment, thoughts about one’s own health, and the feeling of security that participation gives are factors that significantly influence the use of cancer screening. Descriptively, the physician’s recommendation is the strongest factor for participation.

**Conclusion:**

The analyses show that cancer screenings are generally well received by older people in Saxony-Anhalt, but participation in them is not related to health literacy. In keeping with the National Cancer Plan, older people should generally be supported in making an informed decision, for example, through target-group-specific physician education.

## Einleitung/Hintergrund

Die Bundesrepublik Deutschland ist vom demografischen Wandel geprägt [1]. Im Vergleich am stärksten von der Entwicklung betroffen ist das Bundesland Sachsen-Anhalt, dessen Bevölkerung sich zu einem hohen Anteil aus älteren Altersgruppen zusammensetzt^1^ und bundesweit das höchste Durchschnittsalter aufweist [1–3].

Die demografische Entwicklung und die somit steigende Zahl älterer Menschen als vulnerable Gruppe führen, obwohl altersstandardisiert zuletzt teilweise leichte Rückgänge zu erkennen sind [4, 5], zu einer Zunahme der absoluten Zahlen von Krebsneuerkrankungen seit 1970, da diese häufig im höheren Lebensalter auftreten [6–8]. Krebsdiagnosen in einem möglichst frühen und gut behandelbaren Stadium zu diagnostizieren, trägt dazu bei, den Anstieg der Neuerkrankungen und die damit verbundenen Belastungen der gesundheitlichen Versorgung zu begrenzen [4, 9], ebenso wie die Lebensqualität der Betroffenen dank frühzeitiger Diagnose durch schonende Therapien zu erhalten [10]. Im Vergleich zu den wachsenden Neuerkrankungszahlen sind die Sterbefälle demgegenüber lediglich geringfügig angestiegen, da sich die Überlebensaussichten für Krebserkrankte wesentlich verbessert haben [4, 6, 9]. Neben den Fortschritten in der Krebstherapie ist dieser Erfolg auch auf den Ausbau der Krebsfrüherkennungsuntersuchungen (KFU) zurückzuführen, deren Ziel es ist, Krebserkrankungen in einem frühen Stadium zu entdecken und frühzeitig Therapien einzuleiten [4, 6, 9].

Verschiedene Studien [8, 11] dokumentieren, dass die Inanspruchnahme der Früherkennung durch soziodemografische Determinanten, wie das Geschlecht, Alter oder die Bildung, bedingt wird. Des Weiteren stellen eine ärztliche Empfehlung [11–15], die Kostenübernahme durch die Krankenkassen und das durch eine Teilnahme bedingte Sicherheitsgefühl bedeutende Einflussgrößen dar [13]. Auch der Einfluss von Wissen über Krebsfrüherkennung auf die KFU-Inanspruchnahme ist zunehmend Gegenstand wissenschaftlicher Untersuchungen [13–15].

Um sich für oder gegen die Inanspruchnahme einer KFU entscheiden zu können, benötigen die Anspruchsberechtigten entscheidungsrelevante Informationen, wie z. B. über potenziellen Nutzen und Schaden einer Früherkennungsuntersuchung. Das Finden, Verstehen und Beurteilen von gesundheitsrelevanten Informationen sowie das darauf aufbauende Treffen einer Entscheidung (insgesamt als „Gesundheitskompetenz“ – GK – bezeichnet) stellen für die ältere Bevölkerung eine Herausforderung dar [16]. Ältere Erwachsene sind im Umgang mit Gesundheitsinformationen häufig vor Schwierigkeiten gestellt und weisen eine geringere GK als die Allgemeinbevölkerung auf [16].

Nachweise für Assoziationen zwischen der GK und KFU sind bisher begrenzt oder diskrepant [17] und im deutschsprachigen Raum den Autor*innen nicht bekannt. Dennoch wird eine gering ausgeprägte GK als relevante soziale Determinante von Gesundheit mit krebsbedingten Ungleichheiten in Verbindung gebracht [18].

In Bezug auf das ländlich geprägte und in weiten Teilen strukturschwache Bundesland Sachsen-Anhalt liegen für die Bevölkerungsgruppe der älteren Menschen keine aktuellen Erkenntnisse über die Inanspruchnahme von KFU und ihren Determinanten vor. Überdies sind ebenfalls zu weiteren präventiven Leistungen aus Sicht der in Sachsen-Anhalt lebenden Bevölkerung keine fundierten Daten vorhanden.

Ziel der vorliegenden Analyse ist es, die Einflüsse auf die Inanspruchnahme von KFU bei älteren Erwachsenen in Sachsen-Anhalt unter besonderer Berücksichtigung der Gesundheitskompetenz zu eruieren.

## Methoden

Die vorliegenden Untersuchungen wurden im Rahmen einer Querschnittsstudie des Forschungsprojekts „Prävention im Alter Sachsen-Anhalt“ (PrimA LSA; Vorhaben-Nr.: ZS/2019/07/99610, ZS/2020/06/145442) durchgeführt. Das Studiendesign ist an anderer Stelle beschrieben [19]. PrimA LSA orientierte sich an dem Verhaltensmodell der Inanspruchnahme von Gesundheitsdienstleistungen nach Andersen et al. [20].

Anhand eines Fragebogens, bestehend aus insgesamt 56 Fragen, wurden von April bis Juni 2021 die Determinanten, Gründe und Barrieren zur Inanspruchnahme von Präventionsleistungen erhoben (vgl. [19]). Die Zielpopulation umfasste Personen ab 55 Jahren in je 2 städtisch und ländlich geprägten Gemeinden des Bundeslandes Sachsen-Anhalt (*n* = 954). Die Kontaktierung der Teilnehmenden erfolgte auf Basis einer nach Alter und Geschlecht stratifizierten Einwohnermeldeamtsstichprobe. Nach Abzug von verstorbenen potenziellen Teilnehmenden belief sich die bereinigte Bruttostichprobe auf 3654 Personen. Trotz des Verzichts auf eine Nachfassaktion, aufgrund eingekürzter Projektlaufzeit und Ressourcen, konnte eine Rücklaufquote von 25,84 % erzielt werden. Hierbei wurden teilweise ausgefüllte Fragebögen wie vollständige gewertet.

### Teilnahme an KFU und Wissensstand.

Die subjektiv eingeschätzte regelmäßige „Teilnahme an der Krebsfrüherkennung*“* wurde für jede der 6 gesetzlichen Früherkennungsleistungen [21] ermittelt (Mammographie, Stuhltest, Koloskopie, Haut‑, Prostata- und Gebärmutterhals-KFU). Für die multivariablen Analysen wird die Inanspruchnahme dichotomisiert (0 = „bisher noch nie an einer Krebsfrüherkennung teilgenommen“ vs. 1 = „mindestens einmal an einer Krebsfrüherkennung teilgenommen“). Des Weiteren wurden 6 Items zum „Wissen über Krebsfrüherkennung“ verwendet – hierbei handelte es sich um Wissensaussagen, die mit „richtig“, „falsch“ oder „weiß nicht“ zu bewerten waren. Erfragt wurden auch die „Gründe für die Inanspruchnahme einer Früherkennung*“*. Die herangezogenen Items entstammen der 21. Welle des Bertelsmann-Gesundheitsmonitors [13] und wurden entsprechend der Zielfragestellung leicht modifiziert (Tab. 1).

**Table 1:** 

	Häufigkeit (*n*)	Anteil (%)
**Geschlecht (*n*** **=** **949)**		
Männlich	479	50,5
Weiblich	470	49,5
**Alter (*n*** **=** **947)**		
55 bis 64 Jahre	184	19,4
65 bis 74 Jahre	245	25,9
75 bis 84 Jahre	250	26,4
85 Jahre und älter	268	28,3
**Bildung (*n*** **=** **905)**		
Hoch	287	31,7
Mittel	502	55,5
Niedrig	116	12,8
**Gesundheitskompetenzgruppen (*n*** **=** **883)**		
Ausreichend	359	40,7
Problematisch	325	36,8
Inadäquat	199	22,5
**Inanspruchnahme der Krebsfrüherkennung (*n*** **=** **880)**		
Mindestens einmal an einer Krebsfrüherkennung teilgenommen	674	76,6
Bisher noch nie an einer Krebsfrüherkennung teilgenommen	206	23,4
**Gründe für die Inanspruchnahme einer Krebsfrüherkennung (**n **=** **880;** Mehrfachantwort möglich)		
Mein/e Arzt/Ärztin hat mir die Untersuchung empfohlen	395	44,9
Ich habe eine schriftliche Einladung erhalten	133	15,1
Die Krankenkassen übernehmen die Kosten für die Krebsfrüherkennung	141	16,0
Ich habe Angst, an Krebs zu erkranken	131	14,9
Jemand in meiner Familie oder meinem Umfeld ist an Krebs erkrankt oder gestorben	166	18,9
Ich mache mir viele Gedanken um meine Gesundheit	141	16,0
Ich habe gelesen, im Fernsehen gesehen oder Radio gehört, dass es gut ist, wenn man an Krebsfrüherkennungsuntersuchungen teilnimmt	118	13,4
Die Krebsfrüherkennung gibt mir ein sicheres Gefühl	262	29,8
Jemand aus meiner Familie bzw. meinem Umfeld hat mir dazu geraten	50	5,7
Derartige Untersuchungen sind im Bonusprogramm meiner Krankenkasse enthalten	146	16,6
**Wissen über Krebsfrüherkennung (*n*** **=** **954)**		
*Krebsvorsorge und Krebsfrüherkennung sind das Gleiche*		
Falsche Antwort	272	28,5
Weiß nicht/keine Angabe	187	19,6
Richtige Antwort	495	51,9
*Wer zur Krebsfrüherkennung geht, bekommt keinen Krebs*		
Falsche Antwort	148^a^	15,5^a^
Weiß nicht/keine Angabe		
Richtige Antwort	806	84,5
*Wenn bei der Untersuchung kein Krebs entdeckt wurde, kann man sicher sein, dass man gesund ist*		
Falsche Antwort	223	23,4
Weiß nicht/keine Angabe	206	21,6
Richtige Antwort	525	55,0
*Die Untersuchungen zur Krebsfrüherkennung haben auch Risiken*		
Falsche Antwort	205	21,5
Weiß nicht/keine Angabe	312	32,7
Richtige Antwort	437	45,8
*Wenn der Krebs bei der Früherkennung entdeckt wird, ist er zu 100* *% heilbar*		
Falsche Antwort	89	9,3
Weiß nicht/keine Angabe	251	26,3
Richtige Antwort	614	64,4
*Manche bei der Früherkennung entdeckten Tumore sind harmlos und würden lebenslang keine Beschwerden verursachen*		
Falsche Antwort	190	19,9
Weiß nicht/keine Angabe	306	32,1
Richtige Antwort	458	48,0

^a^Die Werte „falsche Antwort“, „weiß nicht/keine Angabe“ werden aufgrund geringer Fallzahlen zusammengefasst

### Gesundheitskompetenz.

Für das Konzept der GK wird die Definition nach Sørensen et al. [22] herangezogen. Gemessen wurde die „Gesundheitskompetenz“ mittels des Health Literacy Survey (HLS)-EU-Q16 [23]. 16 Items sind als direkte Fragen formuliert und ermitteln, inwieweit verschiedene gesundheitliche Situationen und Aktivitäten den Befragungspersonen Schwierigkeiten bereiten. Die 4 Antwortkategorien (1 = sehr schwierig bis 4 = sehr einfach) wurden dichotomisiert und anschließend addiert, um einen individuellen GK-Score zu bilden (0 = schlechtester Wert bis 16 = bestmöglicher Wert). Die Aussagen, die mit „sehr einfach“ sowie „ziemlich einfach“ beantwortet wurden, erhielten den Wert 1. Die Antwortkategorien „ziemlich schwierig“ und „sehr schwierig“ bekamen den Wert 0 zugeordnet [23]. Aus dem Score können Ausprägungen gruppiert werden. Nach Röthlin et al. [23] erfolgte folgende Gruppenzuordnung: 16 bis 13 Punkte = ausreichende GK, 12 bis 9 Punkte = problematische GK, 8 bis 0 Punkte = inadäquate GK.

### Soziodemografie.

Die Items „Geschlecht“, „schulische Bildung“ sowie „berufliche Bildung“ basieren auf den soziodemografischen Standards des Statistischen Bundesamtes [24]. Für den Vergleich mit bildungsstatistischen Merkmalen erfolgte eine Kategorisierung der schulischen und beruflichen Bildung zu Bildungsgruppen mithilfe der CASMIN-Klassifikation [25]. Die Variable „Alter“ wurde aus dem im Fragbogen erfassten Geburtsjahr abgeleitet und in 4 Kategorien unterteilt (Tab. 1).

### Statistische Auswertung.

Die statistische Aufbereitung und Analyse der Daten erfolgten mittels der Statistik- und Analysesoftware IBM SPSS Statistics©, Version 27.0 (International Business Machines Corporation, Armonk, New York, Vereinigte Staaten). Neben der deskriptiven Auswertung erfolgte die Untersuchung des Einflusses der unabhängigen Determinanten auf die Inanspruchnahme der Krebsfrüherkennung anhand einer binär-logistischen Regression in schrittweise komplexer werdenden Modellen. Das Signifikanzniveau wird auf 95 % festgelegt. Eine Imputation fehlender Werte erfolgte nicht. Diese wurden nicht in die Analyse eingeschlossen.

## Ergebnisse

### Beschreibung der Stichprobe

Die Stichprobe umfasste 954 Teilnehmende. Eine Gesamtübersicht der Stichprobendeskription ist der Tab. 1 zu entnehmen. Den größten Anteil der mindestens 55-jährigen Population nahm mit 28,3 % die Altersgruppe „85 Jahre und älter“ ein. Insgesamt haben 76,6 % der Studienpopulation mindestens einmal an einer KFU teilgenommen. Eine ärztliche Empfehlung war mit Abstand der bedeutendste Grund für die Inanspruchnahme einer Früherkennungsuntersuchung. Hingegen war der Rat durch ein Familienmitglied oder aus dem näheren Umfeld der schwächste Beweggrund.

Im Mittel schätzten die Befragten 3,5 von 6 Wissensaussagen richtig ein, wobei über die Hälfte der Studienpopulation mindestens 4 Aussagen korrekt bewertete (Daten nicht gezeigt). Schwierigkeiten bereitete es den über 55-Jährigen zu beurteilen, ob Untersuchungen zur Krebsfrüherkennung Risiken aufweisen.

**Figure 1:**
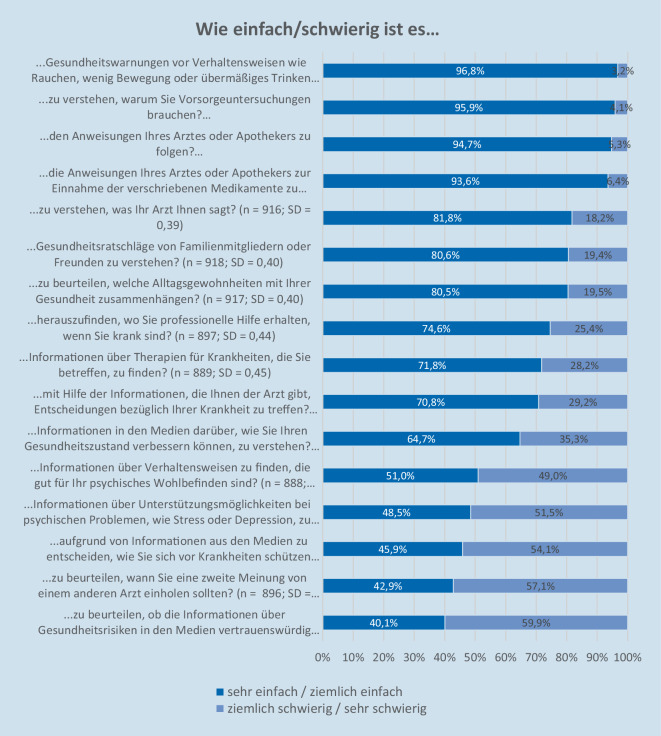

Die Gesundheitskompetenz war bei 40,7 % der älteren Erwachsenen ausreichend ausgeprägt. Für die Mehrheit der Befragten ist es „sehr einfach“ oder „ziemlich einfach“ Gesundheitswarnungen, z. B. zum Thema Rauchen, zu verstehen (96,8 %; Abb. 1). Demgegenüber fällt die Einschätzung der Vertrauenswürdigkeit von Informationen über Gesundheitsrisiken in den Medien oder die Beurteilung der Notwendigkeit einer ärztlichen Zweitmeinung den älteren Sachsen-Anhalter*innen am schwersten (vgl. [26]).

### Multivariable Einflüsse auf die Inanspruchnahme der Krebsfrüherkennung

Für eine differenziertere Betrachtung der einzelnen Einflussfaktoren wurden die unabhängigen Variablen sukzessive in 4 Blöcken (Modelle) in das binär-logistische Regressionsmodell aufgenommen (Tab. 2).

**Table 2:** 

	Modell 1 OR (KI)	Modell 2 OR (KI)	Modell 3 OR (KI)	Modell 4 OR (KI)
	*n* = 836	*n* = 792	*n* = 792	*n* = 792
**Prädiktor**				
*Geschlecht*				
Männlich	Ref.	Ref.	Ref.	Ref.
Weiblich	0,75 (0,54–1,05)	0,73 (0,51–1,02)	0,71 (0,50–1,01)	0,66 (0,42–1,02)
*Alter*				
55 bis 64 Jahre	Ref.	Ref.	Ref.	Ref.
65 bis 74 Jahre	2,06 (1,33–3,19)*	2,13 (1,37–3,32)*	1,94 (1,23–3,07)*	1,99 (1,23–3,24)*
75 bis 84 Jahre	3,43 (2,11–5,59)*	3,26 (1,98–5,36)*	3,01 (1,80–5,03)*	3,41 (1,95–5,96)*
85 Jahre und älter	2,22 (1,36–3,63)*	2,31 (1,39–3,84)*	1,84 (1,06–3,20)*	2,08 (1,14–3,82)*
*Bildung*				
Niedrige Bildung	Ref.	Ref.	Ref.	Ref.
Mittlere Bildung	1,15 (0,65–2,03)	1,24 (0,69–2,23)	1,17 (0,64–2,14)	1,03 (0,55–1,94)
Hohe Bildung	1,16 (0,65–2,08)	1,25 (0,69–2,30)	1,26 (0,67–2,23)	1,06 (0,55–2,03)
*Gesundheitskompetenz*				
Inadäquate Gesundheitskompetenz	n. e.	Ref.	Ref.	Ref.
Problematische Gesundheitskompetenz	n. e.	0,87 (0,54–1,39)	0,87 (0,54–1,40)	0,83 (0,51–1,38)
Ausreichende Gesundheitskompetenz	n. e.	0,88 (0,55–1,40)	0,85 (0,53–1,36)	0,89 (0,54–1,47)
*Krebsvorsorge und Krebsfrüherkennung sind das Gleiche*				
Falsche Antwort	n. e.	n. e.	Ref.	Ref.
Weiß nicht/keine Angabe	n. e.	n. e.	1,00 (0,59–1,70)	1,08 (0,62–1,88)
Richtige Antwort	n. e.	n. e.	1,64 (1,10–2,43)*	1,47 (0,97–2,42)
*Wer zur Krebsfrüherkennung geht, bekommt keinen Krebs*				
Falsche Antwort, weiß nicht/keine Angabe ^a^	n. e.	n. e.	Ref.	Ref.
Richtige Antwort	n. e.	n. e.	0,53 (0,27–1,06)	0,46 (0,23–0,95)*
*Wenn kein Krebs entdeckt wird, kann man sicher sein, dass man gesund ist*				
Falsche Antwort	n. e.	n. e.	Ref.	Ref.
Weiß nicht/keine Angabe	n. e.	n. e.	0,90 (0,51–1,60)	0,78 (0,43–1,41)
Richtige Antwort	n. e.	n. e.	0,98 (0,62–1,54)	1,00 (0,62–1,61)
*Untersuchungen zur Krebsfrüherkennung haben auch Risiken*				
Falsche Antwort	n. e.	n. e.	Ref.	Ref.
Weiß nicht/keine Angabe	n. e.	n. e.	0,82 (0,49–1,39)	0,81 (0,47–1,41)
Richtige Antwort	n. e.	n. e.	0,88 (0,56–1,39)	0,84 (0,52–1,36)
*Wenn Krebs entdeckt wird, ist er zu 100* *% heilbar*				
Falsche Antwort	n. e.	n. e.	Ref.	Ref.
Weiß nicht/keine Angabe	n. e.	n. e.	0,80 (0,38–1,73)	0,71 (0,32–1,57)
Richtige Antwort	n. e.	n. e.	0,73 (0,36–1,46)	0,63 (0,31–1,29)
*Manche bei der Früherkennung entdeckten Tumore sind harmlos und würden lebenslang keine Beschwerden verursachen*				
Falsche Antwort	n. e.	n. e.	Ref.	Ref.
Weiß nicht/keine Angabe	n. e.	n. e.	0,49 (0,28–0,87)*	0,46 (0,26–0,84)*
Richtige Antwort	n. e.	n. e.	0,52 (0,31–0,86)*	0,48 (0,28–0,81)*
*Mein/e Arzt/Ärztin hat mir die Untersuchung empfohlen*				
Nicht gewählt	n. e.	n. e.	n. e.	Ref.
Gewählt	n. e.	n. e.	n. e.	1,22 (0,84–1,77)
*Ich habe eine schriftliche Einladung erhalten*				
Nicht gewählt	n. e.	n. e.	n. e.	Ref.
Gewählt	n. e.	n. e.	n. e.	0,95 (0,56–1,60)
*Die Krankenkassen übernehmen die Kosten für die Krebsfrüherkennung*				
Nicht gewählt	n. e.	n. e.	n. e.	Ref.
Gewählt	n. e.	n. e.	n. e.	0,82 (0,47–1,47)
*Ich habe Angst, an Krebs zu erkranken*				
Nicht gewählt	n. e.	n. e.	n. e.	Ref.
Gewählt	n. e.	n. e.	n. e.	1,05 (0,62–1,80)
*Jemand in meiner Familie bzw. meinem Umfeld ist an Krebs erkrankt oder gestorben*				
Nicht gewählt	n. e.	n. e.	n. e.	Ref.
Gewählt	n. e.	n. e.	n. e.	2,43* (1,45–4,07)
*Ich mache mir viele Gedanken um meine Gesundheit*				
Nicht gewählt	n. e.	n. e.	n. e.	Ref.
Gewählt	n. e.	n. e.	n. e.	1,93* (1,07–3,47)
*Ich habe gelesen, im Fernsehen gesehen oder Radio gehört, dass es gut ist, wenn man an Krebsfrüherkennungsuntersuchungen teilnimmt*				
Nicht gewählt	n. e.	n. e.	n. e.	Ref.
Gewählt	n. e.	n. e.	n. e.	1,17 (0,63–2,18)
*Die Krebsfrüherkennungsuntersuchung gibt mir ein sicheres Gefühl*				
Nicht gewählt	n. e.	n. e.	n. e.	Ref.
Gewählt	n. e.	n. e.	n. e.	1,68* (1,08–2,62)
*Jemand aus meiner Familie bzw. meinem Umfeld hat mir dazu geraten*				
Nicht gewählt	n. e.	n. e.	n. e.	Ref.
Gewählt	n. e.	n. e.	n. e.	2,36 (0,96–5,81)
*Derartige Untersuchungen sind im Bonusprogramm meiner Krankenkasse enthalten*				
Nicht gewählt	n. e.	n. e.	n. e.	Ref.
Gewählt	n. e.	n. e.	n. e.	3,54* (1,74–7,22)
*R*^*2*^ *Modellgüte nach Nagelkerke*	R^2^ = 0,056	R^2^ = 0,057	R^2^ = 0,099	R^2^ = 0,210

Legende: binär-logistische Regression zum Vergleich der KFU-Teilnehmenden und Nicht-Teilnehmenden; abhängige Variable: 1 = „mindestens einmal an einer KFU teilgenommen“ (Modell 1: *n* = 642; Modell 2–4: *n* = 606), 0 = „bisher noch nie an einer KFU teilgenommen“ (Modell 1: *n* = 194; Modell 2–4: *n* = 186); sukzessiver Einschluss der unabhängigen Variablen in Blöcken (Modellen); Modell 1: Einschluss soziodemografischer Merkmale, Modell 2: Einschluss vorheriger Variablen + Gesundheitskompetenz, Modell 3: Einschluss vorheriger Variablen + Wissen über KFU, Modell 4: Einschluss vorheriger Variablen + Gründe für die Inanspruchnahme einer KFU

*KI* 95 % Konfidenzintervall, *n. e.* nicht eingeschlossen, *OR* Odds Ratio, *Ref.* Referenzkategorie

*p < 0,05

^a^Die Werte „falsche Antwort“, „weiß nicht/keine Angabe“ werden aufgrund geringer Fallzahlen zusammengefasst

Der Regressionsanalyse kann entnommen werden, dass das Geschlecht in keinem der Modelle einen signifikanten Einfluss auf die Inanspruchnahme der KFU hatte. Weiterhin war die Bildung in keinem der Modelle mit der KFU-Teilnahme assoziiert. Demgegenüber stieg in allen Modellen im Vergleich zur Referenzkategorie der 55- bis 64-Jährigen die Chance, eine KFU in Anspruch zu nehmen, mit zunehmendem Alter an. Der stärkste Effekt zeigte sich bei der Altersgruppe „75 bis 84 Jahre“ (OR 3,41; KI 95 %: 1,95–5,96). Im Vergleich zur Referenzgruppe erhöhte sich die Chance, die Leistungen der Früherkennung wahrzunehmen, in allen Modellen auch in den Alterskategorien der 65- bis 74-Jährigen (OR 1,99; 1,23–3,24) und mindestens 85-Jährigen (OR 2,08; 1,14–2,81).

Die Regressionsmodelle bilden ab, dass gegenüber einer inadäquaten GK die Chance, eine KFU wahrzunehmen, nicht durch eine höhere GK gesteigert wird. Der Einfluss der problematischen und ausreichenden GK ist im Vergleich in keinem der 4 Modelle signifikant.

Die Wissensdeterminante „Krebsvorsorge und Krebsfrüherkennung sind das Gleiche“, bei welcher im dritten Modell (M) die Chance für eine Inanspruchnahme der Früherkennung durch eine richtige Antwort gesteigert wurde (OR 1,64; 1,10–2,43), ist im vierten Modell nicht mehr signifikant (OR 1,47; 0,97–2,42). Das bedeutet, die erhöhte Teilnahmechance gegenüber einer „falschen“ Antwort besteht nicht mehr. Die korrekte Bewertung der Aussage: „Wer zur Krebsfrüherkennung geht, bekommt keinen Krebs“, minderte innerhalb des vierten Modells die Chance einer Krebsfrüherkennungsteilnahme gegenüber der Referenzgruppe (OR 0,46; 0,23–0,95). Die Ergebnisse der Wissensdeterminante „Manche bei der Früherkennung entdeckten Tumore sind harmlos und würden lebenslang keine Beschwerden verursachen“ bilden sowohl im dritten als auch vierten Modell ab, dass eine korrekte Antwort (M3: OR 0,52; 0,31–0,86; M4: OR 0,48; 0,28–0,81) oder eine Antwort mit „weiß nicht“ (bzw. keine Angabe; M3: OR 0,49; 0,28–0,87; M4: OR 0,46; 0,26–0,84) die Chance der Inanspruchnahme einer Früherkennung gegenüber einer falschen Bewertung verringert.

Die Auswahl folgender Gründe war mit der signifikant erhöhten Chance einer KFU-Inanspruchnahme gegenüber einer Nicht-Wahl dieser Gründe assoziiert: „Jemand in meiner Familie bzw. meinem Umfeld ist an Krebs erkrankt oder gestorben“ (OR 2,42; 1,45–4,07), „Ich mache mir viele Gedanken um meine Gesundheit“ (OR 1,93; 1,07–3,47), „Die Krebsfrüherkennungsuntersuchung gibt mir ein sicheres Gefühl“ (OR 1,68; 1,08–2,62) und „Derartige Untersuchungen sind im Bonusprogramm meiner Krankenkasse enthalten“ (OR 3,54; 1,74–7,22). Letzterer stellte insgesamt den stärksten Einflussfaktor für die KFU-Teilnahme dar.

Die Modellgüte R^2^ verbesserte sich nach Einschluss der GK sowie der Determinanten zur Messung des Wissens über Krebsfrüherkennung im Verhältnis zur ausschließlichen Betrachtung der soziodemografischen Merkmale nur gering (0,056 vs. 0,099). Einen starken Gesamteinfluss auf die Modellgüte üben erwartungsgemäß die Gründe für die Inanspruchnahme einer Früherkennungsleistung aus. Die eingeschlossenen unabhängigen Variablen können im vierten Modell die Varianz in der selbst berichteten Teilnahme an der Krebsfrüherkennung zu ca. einem Fünftel erklären (R^2^ = 0,210).

## Diskussion

Ziel des Beitrags war es, Erkenntnisse zur Nutzung der KFU älterer Erwachsener in Sachsen-Anhalt zu gewinnen. Als wesentliches Ergebnis innerhalb der Befragung kann hervorgehoben werden, dass mehr als drei Viertel der Befragten angeben, bereits an mindestens einer gesetzlichen Früherkennungsleistung teilgenommen zu haben. Ähnliche Daten verzeichnete die erste Erhebungswelle der Studie zur Gesundheit Erwachsener in Deutschland (DEGS1), in welcher 55,5 % die Krebsfrüherkennung regelmäßig und weitere 16,2 % der Frauen und 19,2 % der Männer unregelmäßig in Anspruch nehmen [8].

Das Hauptaugenmerk der vorliegenden Analyse lag auf individuellen Faktoren (vgl. [20]). Gleichwohl ist wichtig zu betonen, dass Sachsen-Anhalt zu weiten Teilen ländlich geprägt ist, strukturschwach und von Ärzt*innenmangel [27] betroffen. Studien zur Untersuchung gynäkologischer Leistungsinanspruchnahme resümierten eine geringere Teilnahme bei ländlichen Wohnorten bspw. aufgrund größerer Entfernungen [28, 29]. Infolgedessen ist denkbar, dass die eingeschränkte Erreichbarkeit von Mediziner*innen in Sachsen-Anhalt die KFU-Inanspruchnahme der Befragung minderte.

Das Finden, Verstehen, Beurteilen und Anwenden von Gesundheitsinformationen (GK) spielt eine bedeutende Rolle, um sich im Gesundheitssystem zurechtzufinden [30]. Häufig wird eine unzureichende GK mit einem mangelnden Bewusstsein für die Bedeutung der Früherkennung [31] und mehreren gesundheitlichen Folgen in Verbindung gebracht, z. B. vermehrte Krankenhausaufenthalte [32]. Die GK war bei 40,7 % der über 55-jährigen Studienpopulation ausreichend, bei 36,8 % problematisch und bei 22,5 % inadäquat ausgeprägt. Die Ergebnisse der Regressionsanalyse zeigen, dass im Vergleich zu einer niedrigen GK die Chance einer KFU-Teilnahme nicht durch eine höhere GK gesteigert wird. Da Personen mit höherer GK ein eher gesundheitsbewusstes Verhalten zeigen [33], kann angenommen werden, dass sie überlegter Vor- und Nachteile der KFU abwägen und sich dabei womöglich bewusst gegen eine Teilnahme entscheiden. Bestehende systematische Übersichtarbeiten weisen auf die Heterogenität der Datenlage hin [17, 34]. Ein Vergleich des Beitrages mit der vorhandenen Evidenz ist erschwert, da ein Großteil der Studien sich häufig lediglich auf eine KFU spezialisiert [12, 35, 36]. Die vorliegende Analyse untersuchte hingegen den Effekt auf die allgemeine Inanspruchnahme und nivelliert damit leistungsspezifische Evidenzlagen. Demnach wäre es denkbar, dass bei einer Untersuchung der spezifischen KFU Effekte durch die GK detektiert werden [17, 34].

Obwohl die GK womöglich die Leistungsinanspruchnahme bedingt – auch wenn diese Annahme im Kontext dieser Untersuchungen nicht bestätigt werden kann, resümieren Studien, dass eine hohe GK allein nicht ausreichend sei, um eine Teilnahme zu erklären, und es andere, wesentlich stärkere Prädiktoren gibt [12, 35]. Im Rahmen der multivariablen Analysen zeigte sich bei Frauen gegenüber Männern keine Erhöhung der Chance einer Früherkennungsteilnahme. Dieses Ergebnis widerspricht einem Großteil der Evidenz. Grundsätzlich gilt es zu berücksichtigen, dass Frauen häufiger ärztliche Leistungen und verhaltenspräventive Angebote in Anspruch nehmen als Männer [37, 38]. Das belegen mehrere Studien zur Inanspruchnahme von KFU [8, 11, 14, 35, 39]. Die Resultate dieser Studien bezogen sich allerdings nicht spezifisch auf ältere Erwachsene, sondern auf alle Leistungsberechtigten.

Die multivariablen Analysen zeigen, dass sich im Vergleich zur Referenzgruppe der 55- bis 64-Jährigen die Chance der Früherkennungsteilnahme in jeder älteren Altersgruppe erhöhte. Dieses Ergebnis reiht sich in die bisherige Evidenzlandschaft ein [8, 11, 12, 14, 15]. Die Zunahme der Inanspruchnahme mit steigendem Alter ist damit zu begründen, dass ältere Menschen häufigeren Arzt‑/Ärztinnenkontakt aufweisen, da körperliche Beschwerden zunehmen [8, 11, 39, 40]. Zudem ist insbesondere bei ausschließlicher Betrachtung der Studienpopulation der älteren Erwachsenen bedeutsam, dass die Darmkrebsfrüherkennung oder Mammographie für die jüngere Altersklasse der untersuchten Population gerade erst an Relevanz gewinnen und ggf. noch unbekannt sind. Die vermehrte Inanspruchnahme mit zunehmendem Alter sollte allerdings differenziert nach Geschlecht betrachtet werden, da die Nutzung der Früherkennungsuntersuchungen zwar bis ins hohe Alter steigt, jedoch je nach Geschlecht früher oder später wieder fällt [11].

Bildung wird im Kontext der Analyse nicht mit der KFU-Inanspruchnahme assoziiert. In der Literatur finden sich hierfür keine einheitlichen Erkenntnisse. Studien erfassen, dass die höheren Bildungsgruppen die Angebote tendenziell eher wahrnehmen [12, 35], resümierten aber zudem, dass dieser Effekt bei Frauen stärker ausgeprägt ist als bei Männern [8, 14]. Die bestehende Literatur schließt allerdings alle Altersgruppen ein. Die Ergebnisse dieser Befragung könnten auch darin begründet liegen, dass lediglich Personen ab 55 Jahren einbezogen wurden. Mutmaßlich wurde ein überwiegender Teil der Studienpopulation in der DDR sozialisiert, dessen Gesundheitssystem explizit den Erhalt, die Förderung und die Wiederherstellung der Gesundheit als politisches Ziel verankert hatte [41], weshalb die Inanspruchnahme von Vorsorge- und Reihenuntersuchungen weitverbreitet war. Diese gesundheitsbezogene Mentalität trägt die untersuchte Zielgruppe ggf. unabhängig vom Bildungsstatus noch in sich, sodass deshalb womöglich keine Zusammenhänge zur Früherkennungsteilnahme vorlagen.

Die Evidenz über direkte Zusammenhänge des Wissens über Krebsfrüherkennung mit der Teilnahme an KFU ist begrenzt. Das Wissen wird häufig selektiv untersucht und es wird angenommen, dass gruppenspezifische Unterschiede (z. B. hinsichtlich des Alters) bestehen, die für die Inanspruchnahme bedeutsam sind [13–15], statistische Zusammenhänge wurden im deutschsprachigen Raum jedoch weniger analysiert. Dem überwiegenden Teil der untersuchten Studienpopulation (84,5 %) war bewusst, dass der Gang zur Früherkennung allein nicht vor Krebs schützt. Diese Aussage wurde auch innerhalb des Gesundheitsmonitors 2012 am häufigsten richtig eingeschätzt [13]. Bei der richtigen Beantwortung der Aussage „Die Untersuchungen zur Krebsfrüherkennung haben auch Risiken“ wies dagegen die Mehrheit der Befragten innerhalb des Gesundheitsmonitors Wissensdefizite auf [13]. Auch in den vorliegenden Untersuchungen verzeichnete diese Determinante den geringsten Anteil an richtigen Antworten, dafür mit Abstand die häufigsten „Weiß-nicht“-Angaben. Angesichts dieser Ergebnisse kann nicht davon ausgegangen werden, dass eine informierte Entscheidung in solchen Fällen möglich ist, da diese unter anderem auf dem Abwägen von Nutzen und Risiken verknüpft mit Präferenzen beruht [13].

Die Chance, an der Krebsfrüherkennung teilzunehmen, war bei Studienteilnehmenden, denen bewusst ist, dass die Früherkennung nicht vor einer Krebserkrankung schützt, signifikant geringer als bei denen, die dies gar nicht oder falsch bewerteten. Auch bei älteren Erwachsenen, die fälschlicherweise die Aussage: „Manche bei der Früherkennung entdeckten Tumore sind harmlos und würden lebenslang keine Beschwerden verursachen“, als „falsch“ einstuften, erhöht sich die Chance der Früherkennungsteilnahme signifikant. Die Ergebnisse lassen selektiv darauf schließen, dass sich Teilnehmende, die ein höheres Wissen über die Krebsfrüherkennung aufweisen, teilweise bewusst gegen die Inanspruchnahme entschieden, da ihnen Nutzen und Risiken der Untersuchungen eher bekannt sind.

Beinahe die Hälfte derjenigen älteren Erwachsenen, die angaben, bereits eine Leistung der Krebsfrüherkennung wahrgenommen zu haben, wählte als wesentlichen Grund zur Inanspruchnahme die ärztliche Empfehlung. Ungefähr 30 % der Befragten empfanden außerdem das sichere Gefühl, welches ihnen eine Früherkennungsteilnahme verleiht, als bedeutenden Motivator. Diese beiden Teilnahmegründe wurden auch in der 21. Welle des Bertelsmann-Gesundheitsmonitors am häufigsten ausgewählt und von älteren Personen signifikant häufiger als bedeutungsvoll eingeschätzt [13]. Dass die ärztliche Empfehlung einer der wichtigsten Prädiktoren bei der Inanspruchnahme einer Früherkennungsuntersuchung ist, bestätigten bereits mehrere Studien [11, 14, 15, 42].

Nach Scheffer et al. [11] sowie Starker et al. [14] sind Familie und Freunde bzw. die Motivation durch den/die Partner*in ausschlaggebend für eine Teilnahme. Ein Rat der Familie oder des näheren sozialen Umfeldes wurde dagegen im Rahmen dieser Befragung am seltensten von den älteren Erwachsenen als Anlass für die Inanspruchnahme genannt (5,7 %). Dies kann darin begründet liegen, dass sich viele Menschen nicht mit Familie oder Freunden über das Thema Krebsfrüherkennung austauschen. In einer Erhebung des AOK-Bundesverbandes gaben z. B. 42 % der Befragten an, „selten“ oder „nie“ im persönlichen Umfeld über Gesundheitsvorsorge oder Vorsorgeuntersuchungen zu sprechen [43]. Demgegenüber wurde der Grund „Jemand in meiner Familie bzw. meinem Umfeld ist an Krebs erkrankt oder verstorben“ als dritthäufigster Grund für eine Inanspruchnahme gewählt. Das zeigt wiederum, dass persönliche Erfahrungen mit einer Krebsdiagnose im sozialen Umfeld für die Untersuchungen zur Krebsfrüherkennung stärker sensibilisieren als eine Empfehlung von Familie oder Freunden.

Scheffer et al. [11] führten 2006 in ihrer systematischen Übersichtsarbeit auf, dass die Entwicklungen der Teilnahmeraten aufgrund der damals durch die Krankenkassen neu eingeführten Bonusprogramme abzuwarten seien. Innerhalb der vorliegenden Analyse hatte die Wahl des Grundes „Derartige Untersuchungen sind im Bonusprogramm meiner Krankenkasse enthalten“ faktisch den stärksten Effekt auf die Chance, Früherkennungsuntersuchungen in Anspruch zu nehmen. Anlass hierfür könnten möglicherweise damit einhergehende Anreize, z. B. monetärer Art, sein. Demgegenüber ist es unerwartet, dass die Kostenübernahme durch die Krankenkasse keinen signifikanten Einflussfaktor darstellte.

Die vorliegenden Projektergebnisse sowie Erkenntnisse aus anderen Modulen der Studie (z. B. aus Fokusgruppendiskussionen mit niedergelassenen Ärzt*innen) wurden in Forschungsnetzwerken des Landes („Autonomie im Alter“) vorgestellt und mit regionalen Kooperationspartnern, wie der Kassenärztlichen Vereinigung Sachsen-Anhalt, Wohlfahrtsorganisationen und Kassen in einem Abschlussworkshop diskutiert. Derartige regelmäßig wiederholte Surveys könnten einen Baustein einer regionalen Gesundheitsberichterstattung darstellen, die verstärkt den Fokus auf bürger*innenbezogene Aspekte der Versorgung und Erreichbarkeit von Versorgungseinrichtungen legt.

### Stärken und Schwächen

An gesundheitsbezogenen Befragungen nehmen tendenziell eher Personen teil, die unabhängig von ihrem Sozial- und Bildungsstatus am Thema Gesundheit interessiert sind (Selektionsbias, „Healthy-Volunteer“-Bias; [7]). Nur ca. 8 % der untersuchten Studienpopulation schätzten ihre Gesundheit als „schlecht“ oder „sehr schlecht“ ein, unter 10 % der Befragten rauchen und ca. 60 % achten „stark“ oder „sehr stark“ auf ihre Gesundheit (Daten nicht gezeigt; Auswertungsdatensatz PrimA LSA). Diese Werte stellen dar, dass die analysierte Zielgruppe eine überwiegend gesunde Stichprobe abbildete, weshalb die Ergebnisse limitiert sind. Überdies resultieren die hohen Krebsfrüherkennungsteilnahmezahlen möglicherweise aus der höheren Bereitschaft der gesundheitsbewussten Population, Früherkennungsuntersuchungen zu nutzen [7, 44]. Zum anderen kann es sein, dass die Erhebung durch eine soziale Erwünschtheit tangiert sowie Unwissenheit und/oder Erinnerungslücken verzerrt wurden [7, 11, 12, 44]. Die Befragung adressierte Erwachsene ab 55 Jahren ohne obere Altersgrenze, weshalb sich teilweise Personen aufgrund von Einschränkungen wie (Multi)Morbidität und Pflegebedürftigkeit gar nicht oder lediglich begrenzt an der Erhebung beteiligen konnten. Aufgrund des explorativen Charakters sowie der Länge der Erhebung ist dies – trotz vorheriger Durchführung eines Pretests – nicht auszuschließen. Die Vergleichbarkeit der Inanspruchnahme im Rahmen dieser Befragung gegenüber anderen Studien ist zudem beschränkt, da andere Erhebungen nicht die regelmäßige oder unregelmäßige Teilnahme ermittelten, sondern die Inanspruchnahme in den letzten 12 oder 24 Monaten u. Ä. [7, 8]. Wiederum setzt das Erfragen einer regelmäßigen und unregelmäßigen Teilnahme voraus, dass die Anspruchsberechtigen wissen, innerhalb welcher Zeitabstände ihnen welche Früherkennungsuntersuchung zusteht. Fazit dessen ist, dass die Selbsteinschätzungen zur Inanspruchnahme grundsätzlich höher liegen als die Abrechnungsdaten [7], weshalb die Ermittlung mittels subjektiver Bewertung nicht als Goldstandard gesehen werden sollte [17].

Die Literatur zum Zusammenhang zwischen KFU-Teilnahmen und der GK im deutschsprachigen Raum ist begrenzt, während internationale Studien überwiegend spezifische KFU analysieren [12, 45]. Die Vergleichbarkeit zu diesen Ergebnissen ist aufgrund von heterogenen Gesundheits‑/Krankenversicherungssystemen und anderen gesundheitlichen Rahmenbedingungen erschwert. Zudem können Befunde zumeist nicht generalisiert werden, da sie andere Instrumente zur Messung der GK einsetzten (z. B. REALM), Teilnehmende mehrheitlich in Kliniken rekrutierten, ethnische Minderheiten (z. B. hispanische Frauen, Personen mit niedrigem sozioökonomischen Niveau) oder zu kleine Stichproben analysierten [17, 34, 46, 47].

### Fazit

Um ihre Gesundheit zu erhalten, ist es für ältere Menschen erforderlich, gesundheitsrelevante Entscheidungen treffen zu können, wie solche zur Inanspruchnahme einer Präventionsleistung wie der Krebsfrüherkennung. Obwohl die Sterberate an Krebs in Ländern mit lange bestehenden und gut organisierten Früherkennungsprogrammen deutlich niedriger ist als in Ländern ohne solche Angebote [5], ist deren Inanspruchnahme noch immer ein kontrovers diskutiertes Thema [10, 44]. Dabei ist es relevant, ob Anspruchsberechtigte eine Früherkennungsteilnahme deshalb ablehnen, weil ihnen die Alternativen zur Entscheidungsfindung fehlen oder weil sie sich aufgrund einer individuellen Beurteilung der Evidenz sowie ihrer eigenen Präferenzen bewusst dagegen entscheiden [13]. Für eine präferenzbasierte Entscheidung ist nicht zuletzt die Aufklärung durch ärztliche Leistungserbringende bedeutungsvoll. Die Ergebnisse unterstreichen, dass diese rein deskriptiv eine zentrale Schlüsselposition für die älteren Erwachsenen darstellen und sie ggf. Anstöße benötigen, um eine Inanspruchnahme abzuwägen. Zudem unterstützen eine höhere GK, aber auch die Verfügbarkeit und ein grundsätzlich erleichterter Zugang zu qualitativ hochwertig aufbereiteten, evidenzbasierten Informationsmaterialien das Treffen informierter Entscheidungen und ermöglichen es, eine aktive Rolle im Versorgungsprozess einzunehmen.

In Anbetracht dessen, dass es starke Belege für einen Zusammenhang zwischen GK und anderen Gesundheitsergebnissen gibt [32, 48, 49], sind weitere Forschungen zum Thema Krebsfrüherkennung und GK insbesondere im deutschsprachigen Raum sinnvoll, um die Versorgungsangebote sowie deren Kommunikation adaptieren zu können.

## Footnotes

1 Nach eigenen Berechnungen – basierend auf Daten des Statistischen Jahrbuchs Sachsen-Anhalt [1, 2] – lässt sich der Anteil an mindestens 55-Jährigen auf rund 44,7 % der Gesamtbevölkerung des Bundeslands beziffern (Stichtag: 31.12.2021).
